# A new species of *Gadirtha* Walker (Nolidae, Eligminae): a proposed biological control agent of Chinese tallow (*Triadica sebifera* (L.) Small) (Euphorbiaceae) in the United States

**DOI:** 10.3897/zookeys.382.6600

**Published:** 2014-02-19

**Authors:** Michael G. Pogue

**Affiliations:** 1 Systematic Entomology Laboratory, PSI, Agricultural Research Service, U.S. Department of Agriculture, c/o Smithsonian Institution, P.O. Box 37012, NMNH, MRC-168, Washington, DC 20013-7012, USA

**Keywords:** China, Taxonomy, new species, biological control, larva, pupa

## Abstract

*Gadirtha fusca* **sp. n.**, is described from Guangxi Province, China. *Gadirtha fusca* differs in forewing color and pattern, male and female genitalia, and in larval pattern from all other species of *Gadirtha*. *Gadirtha fusca* has been evaluated as a potential biological control agent for Chinese tallow (*Triadica sebifera* (L.) Small, Euphorbiaceae) in the southeastern United States. Adult, male and female genitalia, larva, and pupa are described, illustrated, and compared with *Gadirtha impingens* Walker.

## Introduction

The genus *Gadirtha* Walker was revised by Holloway (2003) and included three species; the type species *Gadirtha impingens* Walker, *Gadirtha pulchra* Butler, and *Gadirtha inexacta* Walker. *Gadirtha pulchra* and *Gadirtha impingens* are the most widespread. *Gadirtha pulchra* ranges from the Indian Subregion, to the Ryukyu Islands in Japan, and Thailand, Singapore, New Guinea, and Queensland, Australia; *Gadirtha impingens* ranges from northern India and southern China to Queensland, the Bismarcks, and Solomons, (Holloway 2003) and from Honshu, Shikoku, Kyushu, and Tsushima in Japan (http://www.jpmoth.org). *Gadirtha inexacta* is found in northern India and Burma, and *Gadirtha fusca* in southern China.

A molecular phylogeny of Nolidae used eight genes to produce a stable phylogeny that consisted of eight strongly supported subfamilies (Zahiri et al. 2013). Holloway (2003) originally placed *Gadirtha* in the Collomeninae, but the results of Zahiri et al. (2013) moved all of the Eurasian genera formerly associated with Collomeninae to the subfamily Eligminae. Holloway (2011) preempted the placement of *Gadirtha* in Eligminae based on characters outlined in Zahiri et al. (2013). Members of Eligminae have an elongate and narrow forewing and in some genera the uncus is absent in the male genitalia. Species of *Gadirtha* have an elongate and narrow wing and the uncus is replaced by stiff, hairlike setae.

*Gadirtha fusca* is being considered as a potential biocontrol agent against Chinese tallow (*Triadica sebifera* (L.) Small, Euphorbiaceae) in the southeastern United States and formal description is critical to this process. Once *Gadirtha fusca* is introduced it will be the largest Nolidae in North America and can easily be distinguished by its elongate forewing with a truncate apex, dark gray forewing ground color with reduced pattern, and a dark gray hind wing. These characters will also distinguish it from other *Gadirtha* species in Asia. This paper describes the last instar, pupa, adult, and male and female genitalia.

## Methods and materials

Images of adults and genitalia were taken with a digital camera, macro lenses, and a pulsed xenon flash. Images were enhanced with Adobe Photoshop® CS4.

Genitalia dissections follow Pogue (2002), except the genitalia were mounted in euparol. Vesica was inflated with 99% isopropyl alcohol and stained in Orcein.

Comparisons of forewing ground color and pattern, hind wing color, and male and female genitalic structures were compared with all species of *Gadirtha* using Holloway (2003). The male genitalia of *Gadirtha fusca* most closely resembled *Gadirtha impingens*, but differences are illustrated by comparing Figures 5 and 6.

Material used in this study is deposited in the following institutions: The Natural History Museum, London (BMNH), Canadian National Collection, Agriculture Canada, Ottawa, Canada (CNC), and National Museum of Natural History, Smithsonian Institution, Washington, DC (USNM).

## Key to species of *Gadirtha*

**Table:** 

1	Forewing with a distinct, thin, black postmedial line from costa slightly excurved then abruptly angled to tornus; hind wing white with a narrow dark gray shading along outer margin, wing veins highlighted with dark gray (see plate 9, fig. 25 in Holloway (2003))	*Gadirtha pulchra*
–	Forewing with postmedial line indistinct, consisting of only a few black scales; hind wing white with broad gray marginal band or completely gray	2
2	Forewing dark gray; hind wing dark gray	*Gadirtha fusca* sp. n.
–	Forewing pale gray to brownish gray; hind wing white basally with a broad dark marginal band	3
3	Male genitalia with costal arm of valve slightly curved with a dorsal triangulate projection near apex (see fig. 502 in Holloway (2003))	*Gadirtha inexacta*
–	Male genitalia with costal arm bent at a 90° angle (Fig. 5)	*Gadirtha impingens*

## Descriptions

### Gadirtha fusca

Pogue sp. n.

http://zoobank.org/9AB88C1D-C3DD-4113-8B75-94656AFDFAA8

http://species-id.net/wiki/Gadirtha_fusca

Figs 1–2 , 5 , 7 , 9 , 11 [Fig F6] [Fig F7] –20

#### Type-locality.

China, Guangxi Province, 4.4 km NW Yangshuo, 24.79833°N, 110.45067°E.

#### Type-specimen.

Holotype male, Original label: “China, Guangxi Province, 4.4 km NW Yangshuo, 24.79833°N, 110.45067°E, 8 June 2012” “Reared from leaf *Triadica sebifera* 10-Sep.-2012 from IPRL colony” USNM ENT 00149216” “HOLOTYPE / *Gadirtha fusca*/ Pogue” [red printed label]. Deposited in USNM.

#### Paratypes.

3 males and 4 females same data as holotype; 2 male genitalia slides USNM 136482, 136485; 2 female genitalia slides USNM 136483, 136484. 5 males and 5 females same data as holotype, from IPRL colony May 2013. USNM, BMNH, CNC.

#### Diagnosis.

**Figures 1–4.**
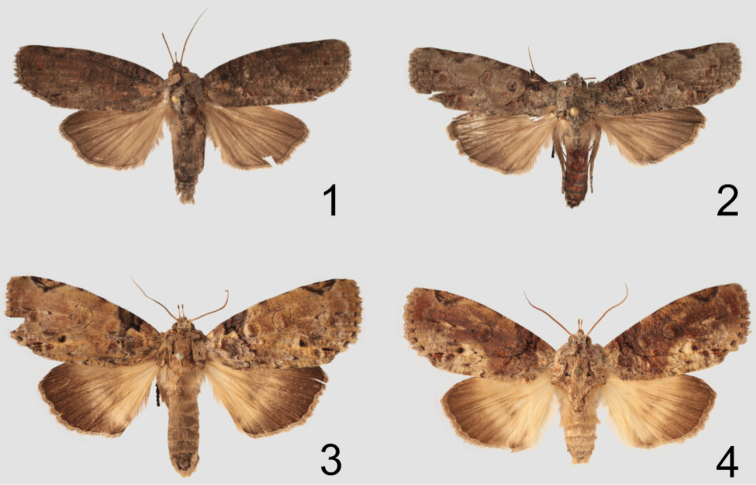
Adults of *Gadirtha* species. **1** *Gadirtha fusca* sp. n., Holotype, male **2** *Gadirtha fusca* sp. n., female **3** *Gadirtha impingens* Walker, male **4** *Gadirtha impingens* Walker, female.

**Figures 5–6.**
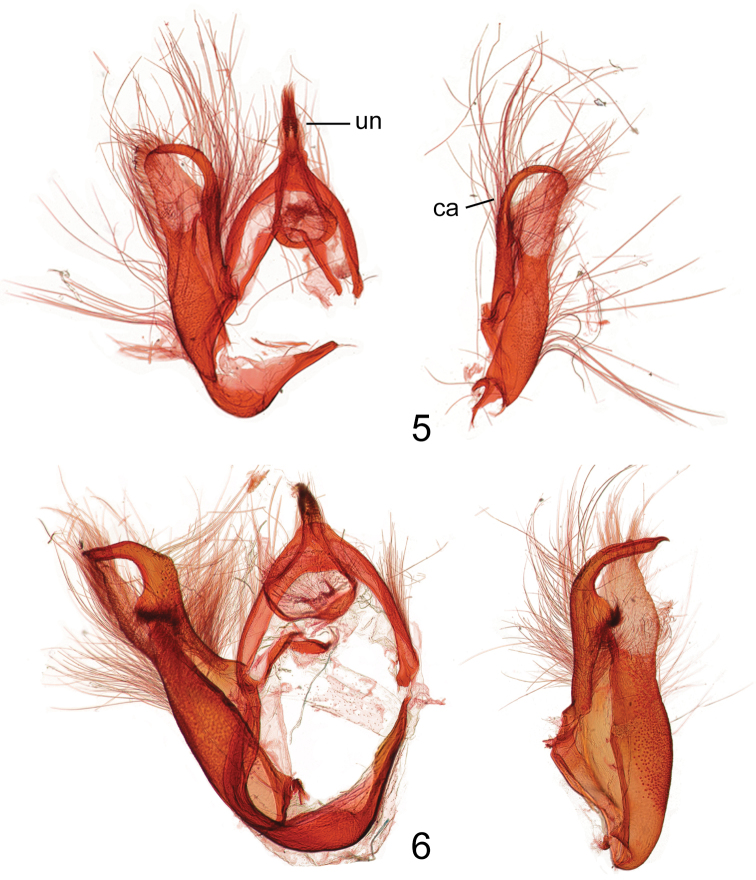
Male genitalia of *Gadirtha* species; **un** uncus **ca** costal arm of valve. **5** *Gadirtha fusca* sp. n. **6** *Gadirtha impingens* Walker.

**Figures 7–8.**
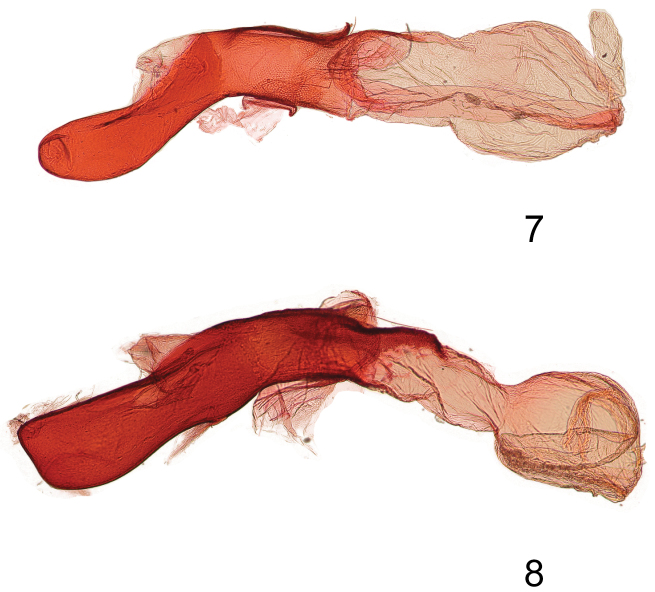
Aedeagi of *Gadirtha* species. **7** *Gadirtha fusca* sp. n. **8** *Gadirtha impingens* Walker.

**Figures 9–10.**
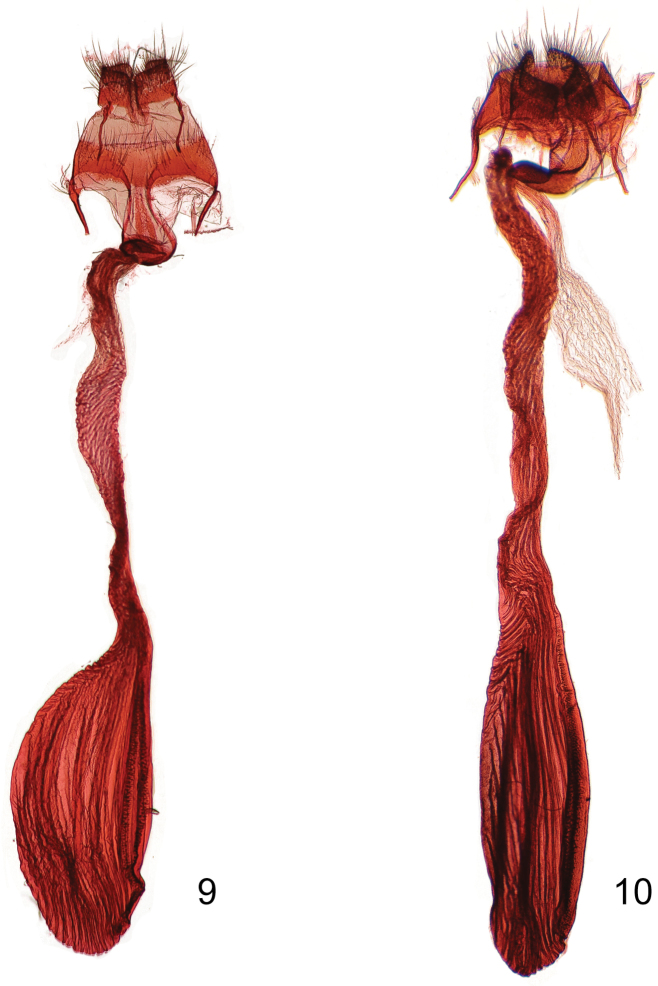
Female genitalia of *Gadirtha* species. **9** *Gadirtha fusca* sp. n. **10** *Gadirtha impingens* Walker.

In the male genitalia *Gadirtha fusca* is most closely related to *Gadirtha impingens* as they share the same 90° angle in the apex of the costa in the valve (Figs 5–6). The aedeagus is abruptly bent medially in *Gadirtha fusca* (Fig. 7) and curved beyond the mid-point in *Gadirtha impingens* (Fig. 8). In *Gadirtha pulchra* and *Gadirtha inexacta* the costal arm of the valve is slightly bent, not curved at a 90° angle. The costal arm of the valve in *Gadirtha inexacta* bears a dorsal triangulate projection near its apex (see fig. 502 in Holloway (2003)) and this projection is absent in *Gadirtha pulchra* see fig. 500 in Holloway (2003). In the female genitalia the ostium bursae in *Gadirtha fusca* is membranous internally with a thin, sclerotized outer margin shaped like an up-side-down conventional incandescent light bulb (Fig. 9); compared to a heavily sclerotized ostium bursae that is strongly curved ventrally (Fig. 10) in *Gadirtha impingens*. *Gadirtha fusca* cannot be confused with any of the other three species of *Gadirtha* with its gray forewings, subdued pattern, and solid dark gray hind wings (Figs 1–2). Forewing ground color is brown to brownish gray in *Gadirtha impingens* with distinct black costal spots (Figs 3–4), contrasting with the dark gray forewing and faint costal spots in *Gadirtha fusca*. Hind wing in *Gadirtha fusca* is dark gray and white basally and dark brown distally in *Gadirtha impingens*. Only *Gadirtha fusca* and *Gadirtha impingens* are distributed in China.

Larvae have a pale green to yellow-green ground color with elongate black and white setae. Pattern differences are distinct between species. *Gadirtha fusca* has a distinct, wide black dorsal stripe with large, ovate, black spots on A1, A2, and A8 (Figs 13–14). In *Gadirtha impingens*, the dorsal black stripe can be absent or if present, a thin line on each segment that is not contiguous; black ovate spots on A1 and A2 are smaller than in *Gadirtha fusca* and a small black round spot on A8 (http://jpmoth.org/Nolidae/Eligminae/Gadirtha_impingens.html). In *Gadirtha pulchra* the black spots are ringed with blue on T2–A4, with the largest spots on T3 and A1 (colour plate 12, fig. F in Murphy 1990).

#### Description.

**Adult** (Figs 1–4): **Male.** *Head* – labial palp extends well above head, apical segment with slightly bulbous apex, dorsal surface adjacent to eye black, becoming pale gray with apical third dark gray, apex white, ventral surface white; frons and vertex gray; antenna ciliate and bare ventrally, white scales dorsally. *Thorax* – patagium grayish brown, a curved black line from middle to posterior margin; tegulum with pale gray scales tipped white, a few tipped black, black band of scales just proximal to apex; scales of protibia narrow, gray tipped white, greatly expanded laterally and ventrally almost to last tarsal segment, two vertical black spots medially that continue onto tarsal segments 1–4, tarsi concolorous with protibia with laterally expanded scales; mesothoracic tibia concolorous with protibia, scales expanded laterally less than in protibia and expanded ventrally to basitarsus, tarsi black dorsally, a few brown scales laterally, white ventrally; metathoracic tibia cream colored, tarsi cream colored with black basal bands on segments 2–4. Forewing length 18.5–22.4 mm; ground color brownish gray; varying amounts of indistinct rufous areas distributed over forewing; costa with rectangulate dark gray basal spot and a fainter dark gray triangulate spot proximal to apex; antemedial line black, very thin, angulate from R vein to anal vein; medial line absent; orbicular spot obscure, a round area consisting of a few pale scales bordered either distally or proximally by a crescent-shaped band of dark gray scales; reniform spot round with a slightly produced apex along distal margin, thinly outlined in black, medially a vertical bar shape to ovate spot, rufous to dark gray; postmedial line very thin, black, indistinct, irregular in shape, excurved; black distal line at apex of vein CuA2; terminal line a series of black dashes between wing veins; outer margin angled at tornus to posterior margin; posterior margin a narrow white line with closely spaced black vertical lines, this line can be variable in extent and intensity. Hind wing dark gray. *Abdomen* – Dark gray. *Genitalia* (Figs 5, 7) – Uncus slightly sclerotized with stiff, hair-like setae at apex; subscaphium well developed, a wide U-shape with bottom of U broad; valve bifurcate, costa curved ventrally to just beyond apex of valve, cucullus lightly sclerotized; corona absent; saccus a broad U-shape; aedeagus short, angulate medially, vesica an elongate sac, slightly bulbous apically. **Female.** *Head* – Antenna not ciliate. *Thorax* – Similar to male except forewing length 20.9–23.1 mm; ground color pale brownish gray; antemedial line irregular and minutely wavy extending from costa almost to posterior margin; orbicular spot more pronounced than in male, a central area of pale scales surrounded by a dark gray crescent-shaped border usually disto-ventrally; a subapical elongate triangular spot on costa with two short black dashes near apex of spot. *Genitalia* (Fig. 9) – Papillae anales rectangulate, setose, slightly sclerotized; ostium bursae bulb-shaped, narrow at exit of eighth tergite, then bulbous basally; ductus bursae short, sclerotized, and quadrate at exit from ostium bursae, remainder elongate, membranous slightly widens to elongate corpus bursae; signum an elongate, crenulate ribbon almost length of corpus bursae.

**Figures 11–12.**
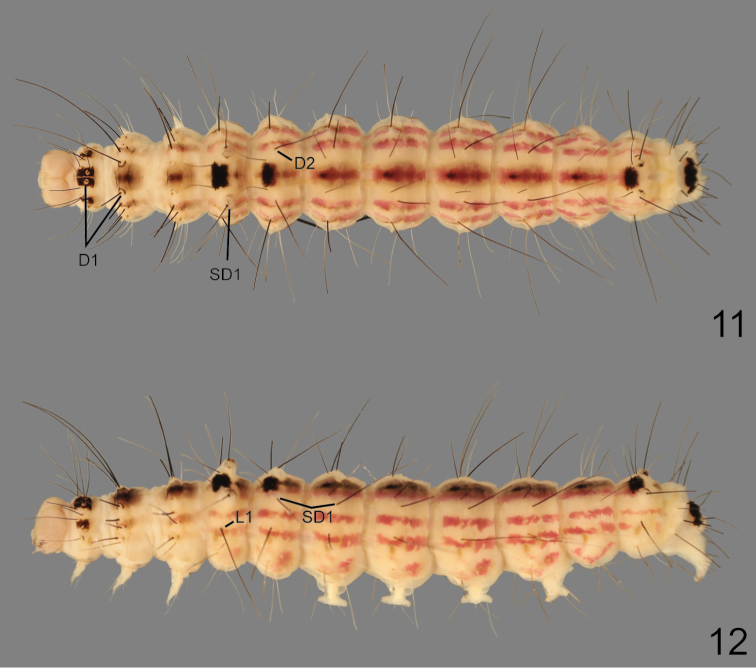
Larva of *Gadirtha fusca* sp. n. (preserved in alcohol). **11** dorsal **12** ventral.

**Figures 13–14. F6:**
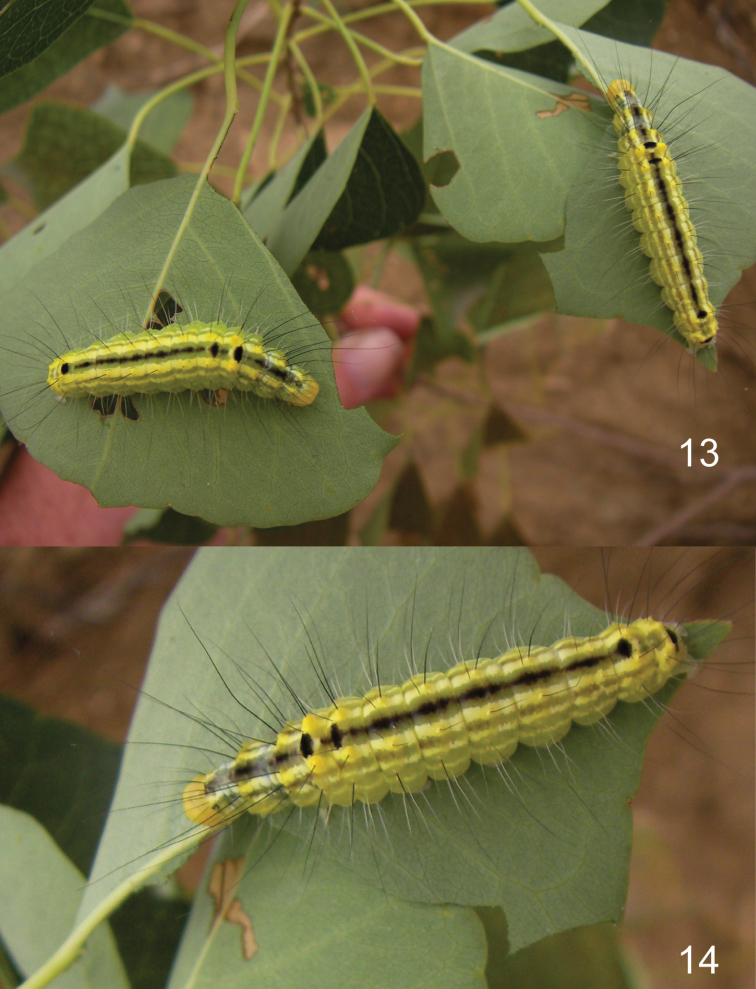
Larva of *Gadirtha fusca* sp. n. **13** on Chinese tallow **14** on Chinese tallow.

**Larva** (Figs 11–14): Length: 28.0–33.5 mm (*n* = 6). **Coloration and pattern.** *Head* – Yellow. *Thorax* – T1 with medial rectangular black spots surrounding D1, D1 pinnacula yellow, irregular shaped black spot between XD2 and SD1, black spinules distal to all black spots; T2 and T3 with patch of black spinules between D1s, between D2 and SD2, distal to L group, and dorsal to SV group. *Abdomen* – yellow; dorsal line distinct black, ovate spots on A1, A2, and A8, remainder of segments with small round spots, spiculate; subdorsal, lateral, and spiracular lines pale green, spiculate; A10 with D1s surrounded by large, irregular shaped black spot, pinnacula yellow. **Morphology.** *Head* – Front flat; hypognathous; cutting edge of mandible with 3 shallow teeth, dorso-medial internal surface with large molar-like tooth, round and peg-shaped; epicranial suture elongate; epicranial notch moderately emarginated dorsally; F1, AF1 equal in length, AF2 longer; C1 shorter than C2; P1 dorsal to AF2, P2 inline with and dorsal to P1, shorter than P1; L1 fine, shorter than A3; S2 distal to stemma 1, shorter than S1; S1 ventral to stemma 3; stemmata 1–3 large and equal in width, larger than stemmata 4–6; spinneret cylindrical, equal in length to labial palp. *Thorax* – Spiculate; verrucae absent; T1 with D1, D2, XD1, and XD2 elongate, black; SD1 and SD2 on prothoracic shield, pale, less than half length of other setae; single prespiracular seta; SV1 and SV2 on same pinnacula; ventral projection present. T2 and T3 with D1, D2, SD1, and SD2 elongate, black on separate, conical pinnacula; L1 and L2 on same pinnacula, black (L1 pale on T3), L2 longest, L3 short, pale, and hairlike; SV1 and SV2 elongate, white, on same pinnacula. *Abdomen* – Spiculate; A1 with D1, D2, SD1, and L1, elongate, black; D2 on very large conical pinnacula; SD2 white, hairlike, directly caudal to spiracle; L1 brown, posterior to spiracle, L2 and L3 white on separate pinnaculae; SV1 and SV2 present. A2 with D1, D2, SD1, L1, elongate, black; D2 on conical pinnacula, but smaller than on A1; rest of setae as in A1 except L1 posterior and ventral to spiracle. A3–A6 with D1, D2, and SD1 elongate, black; SD2 white, hairlike; L2 and L3 white on separate pinnaculae; 3 SV setae, white; crochets uniordinal mesoseries. A7–A8 with D1 and D2 black, L1 white on A7 and black on A8, elongate; SD2 caudal to spiracle, white, hairlike; on SV seta. A9 with D1, D2, SD1, and L1, elongate, black; L and SV group absent; A9 appears to fuse with A8 ventrally. A10 with D1 and SD1 brown, elongate; SD2 pale brown, elongate; D2 white, elongate; SV1, L2 and L3 brown, elongate; 4 SV setae white; crochets uniordinal mesoseries.

**Figures 15–17. F7:**
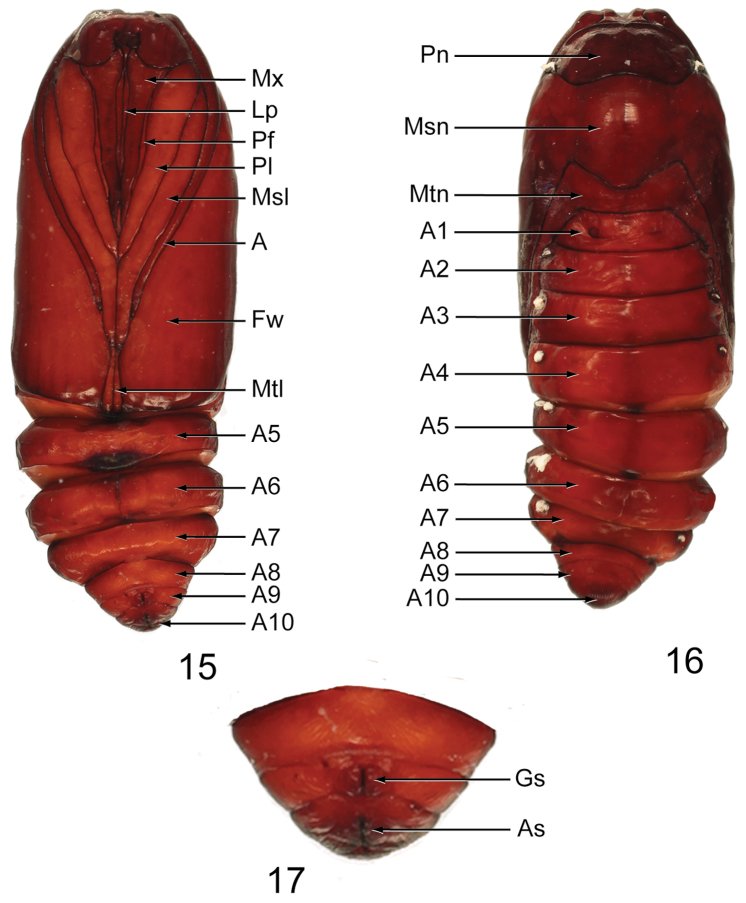
Male pupa of *Gadirtha fusca* sp. n. (preserved in alcohol). **15** dorsal **16** ventral **17** closeup of A8–A10 (**A** antenna; **As** anal suture; **Fw** forewing; **Gs** genital suture; **Lp** Labial palp; **Msl** mesothoracic leg; **Msn** mesonotum; **Mtl** metathoracic leg; **Mtn** metanotum; **Mx** maxillae; **Pf** prothoracic femur; **Pl** prothoracic leg; **Pn** pronotum).

**Figures 18–20.**
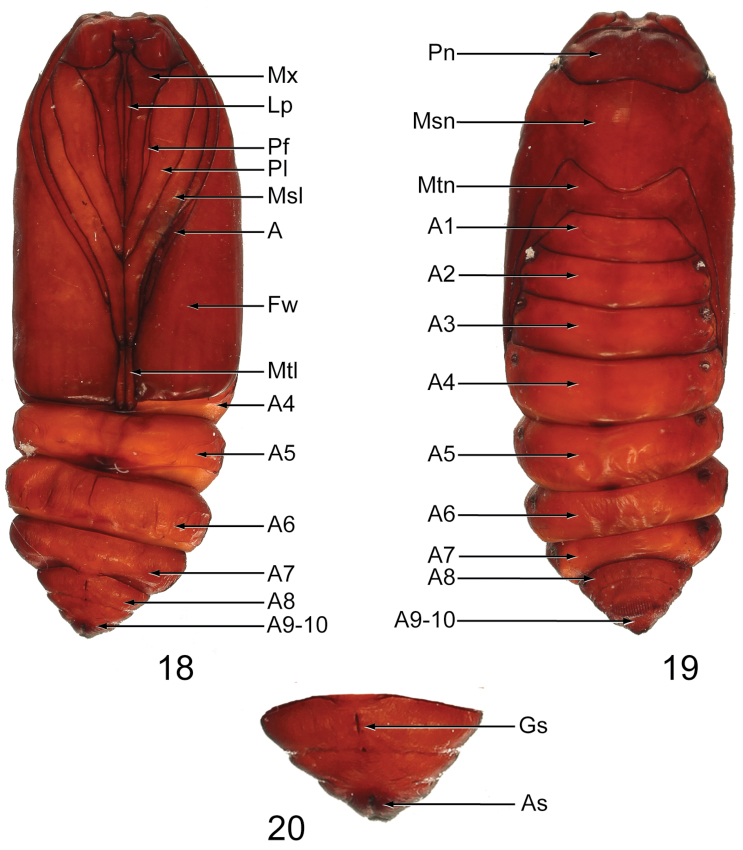
Female pupa of *Gadirtha fusca* sp. n. (preserved in alcohol). **18** dorsal **19** ventral **20** closeup of A8–A10 (**A** antenna; **As** anal suture; **Fw** forewing; **Gs** genital suture; **Lp** Labial palp; **Msl** mesothoracic leg; **Msn** mesonotum; **Mtl** metathoracic leg; **Mtn** metanotum; **Mx** maxillae; **Pf** prothoracic femur; **Pl** prothoracic leg; **Pn** pronotum).

**Pupa** (Figs 15–20): Length: 18.8–19.0 mm in male (*n* = 3) (Figs 15–17), 18.8–19.2 mm in female (*n* = 3) (Figs 18–20). Obtect; adecticous; smooth, dark brown; labial palpus (Lp) extends from clypeus to approximately 0.7 × length of maxillae (Mx); prothoracic leg (Pl) approximately 1.16 × length of maxillae; prothoracic femora (Pf) present, narrow, shorter than maxillae; maxillae short, approximately half the length from clypeus to forewing apex; mesothoracic leg (Msl) approximately 0.8 × length from clypeus to forewing apex; antenna (A) shorter than mesothoracic leg; metathoracic leg (Mtl) visible between mesothoracic leg and apex of forewing; segments A8–A10 separate in male A9–A10 fused in female; genital suture on A9 in male set between two bumps and on A8 in female; anal suture on A10; cremaster absent.

#### Etymology.

The specific epithet refers to the dark grayish brown ground color of the forewing.

#### Biology.

*Gadirtha fusca* overwinter as eggs on leaves and branches of Chinese tallow and hatch in May. Larvae feed on leaves and complete six instars in 15 days and can cause extensive defoliation, especially during the last three instars. There can be 4–5 generations per year in Hubei Province (Wang et al. 2012).

#### Distribution.

East central and southeastern China.

#### Discussion.

It is curious why *Gadirtha fusca* was misidentified in the biocontrol literature as *Gadirtha inexacta* (Wang et al. 2012). In reviewing Holloway (2003), *Gadirtha inexacta* does not occur in China and the life history is unknown. The more obvious misidentification would be with *Gadirtha impingens*, which does occur in China, larvae are known to feed on species of Euphorbiaceae, and morphology of the male genitalia are similar.

## Supplementary Material

XML Treatment for Gadirtha fusca
